# Culturally Adapting the IPROACTIF Intervention for Spanish-Speaking Latinx Adults With Hypertension and Diabetes

**DOI:** 10.1093/geroni/igaf122.1385

**Published:** 2025-12-31

**Authors:** Dalmina Arias, Mansha Mirza

**Affiliations:** University of Illinois Chicago, Chicago, Illinois, United States; University of Illinois Chicago, Chicago, Illinois, United States

## Abstract

Diabetes is highly prevalent in the U.S. Latino population, with adults over age 18 having nearly twice the prevalence of diabetes compared to non-Hispanic whites. Likewise, hypertension is an important comorbid condition with significantly higher prevalence in the Latino community compared to non-Hispanic whites. While several effective self-management interventions have been developed to improve health outcomes of older adults with diabetes, few have been culturally adapted to address the specific needs of older Latinos. This study adapts the Integrated PRimary care and Occupational therapy for Aging and Chronic disease Treatment for Independence and Functioning (IPROACTIF) intervention for Spanish-speaking Latino older adults with hypertension and diabetes using the Ecological Validity Framework. We interviewed 16 older Latinos residing in the Chicago Area who were living with diabetes and/or hypertension about their experiences managing their chronic conditions and the role of healthcare services. Stage two of this project will interview service providers about their experiences with older Latino populations. Through focus groups and thematic analysis, we identify barriers to health management and adapt the intervention’s language, content, and context. Preliminary results have identified understanding of medication, insurance and continuity of care, and language as barriers to self-management of chronic conditions for older Latinos. We these barriers in mind, we work to adapt the IPROACTIF to patient experiences and a culturally tailored intervention. This work addresses health disparities by improving chronic disease management in Latino older adults.

